# NF-κB mediates Gadd45β expression and DNA demethylation in the hippocampus during fear memory formation

**DOI:** 10.3389/fnmol.2015.00054

**Published:** 2015-09-16

**Authors:** Timothy J. Jarome, Anderson A. Butler, Jessica N. Nichols, Natasha L. Pacheco, Farah D. Lubin

**Affiliations:** Department of Neurobiology, University of Alabama at BirminghamBirmingham, AL, USA

**Keywords:** nuclear factor kappa B, hippocampus, DNA demethylation, Gadd45, memory

## Abstract

Gadd45-mediated DNA demethylation mechanisms have been implicated in the process of memory formation. However, the transcriptional mechanisms involved in the regulation of *Gadd45* gene expression during memory formation remain unexplored. NF-κB (nuclear factor kappa-light-chain-enhancer of activated B cells) controls transcription of genes in neurons and is a critical regulator of synaptic plasticity and memory formation. *In silico* analysis revealed several NF-κB (p65/RelA and cRel) consensus sequences within the *Gadd45β* gene promoter. Whether NF-κB activity regulates *Gadd45* expression and associated DNA demethylation in neurons during memory formation is unknown. Here, we found that learning in a fear conditioning paradigm increased *Gadd45β* gene expression and *brain-derivedneurotrophic factor (BDNF)* DNA demethylation in area CA1 of the hippocampus, both of which were prevented with pharmacological inhibition of NF-κB activity. Further experiments found that conditional mutations in *p65/RelA* impaired fear memory formation but did not alter changes in *Gadd45β* expression. The learning-induced increases in *Gadd45β* mRNA levels, Gadd45β binding at the *BDNF* gene and *BDNF* DNA demethylation were blocked in area CA1 of the *c-rel* knockout mice. Additionally, local siRNA-mediated knockdown of *c-rel* in area CA1 prevented fear conditioning-induced increases in Gadd45β expression and *BDNF* DNA demethylation, suggesting that c-Rel containing NF-κB transcription factor complex is responsible for *Gadd45β* regulation during memory formation. Together, these results support a novel transcriptional role for NF-κB in regulation of Gadd45β expression and DNA demethylation in hippocampal neurons during fear memory.

## Introduction

The formation of long-term memories requires dynamic changes in gene transcription and protein translation in neurons (Johansen et al., 2011; Jarome and Helmstetter, 2013). Over the last decade numerous studies have implicated epigenetic mechanisms, which regulate transcription without modifying the underlying gene sequence, in this memory consolidation process (Stefanko et al., 2009; Gupta et al., 2010; Gräff and Tsai, 2013; Jarome et al., 2014; Kwapis and Wood, 2014). Of the epigenetic mechanisms identified, DNA methylation has become particularly attractive due to its potential to regulate gene expression across the lifespan (Roth et al., 2009). Active DNA methylation regulates expression of several memory-associated genes during the memory consolidation process and manipulation of DNA methyltransferase (DNMT) activity impairs memory for a variety of behavioral tasks (Miller and Sweatt, 2007; Miller et al., 2008, 2010; Feng et al., 2010; Maddox et al., 2014). In addition to the strong evidence that exists for *de novo* DNA methylation during memory formation, recent studies have indicated active DNA demethylation in the hippocampus and neocortex during memory formation and extinction (Kaas et al., 2013; Rudenko et al., 2013). This suggests that the memory consolidation process requires both gene activation and repression mediated by DNA methylation and demethylation mechanisms, respectively. However, very little is known about how DNA demethylation is regulated during the memory consolidation process.

The Growth arrest and DNA damage-inducible 45 (Gadd45) family of proteins are stress sensor genes that have been implicated in active DNA demethylation (Barreto et al., 2007; Niehrs and Schäfer, 2012). This family of proteins consists of the alpha, beta and gamma isoforms whose expression is dynamically altered as a function of learning (Leach et al., 2012). The Gadd45β isoform has been shown to mediate gene-specific DNA demethylation in the dentate gyrus following seizure and during neurogenesis (Ma et al., 2009) and one of the target genes is brain-derived neurotrophic factor (BDNF), which undergoes promoter-specific DNA demethylation in the hippocampus during memory consolidation (Lubin et al., 2008). Further, knockout of Gadd45β alters long-term potentiation and memory retention for hippocampus-dependent tasks (Leach et al., 2012; Sultan et al., 2012), supporting a role for the Gadd45 family of proteins in synaptic plasticity and memory formation (Sultan and Sweatt, 2013). However, to date, it is unknown how Gadd45 expression and its DNA demethylation activity is regulated in the hippocampus during memory consolidation.

The Nuclear Factor Kappa B (NF-κB) transcription factor exists as a homo- or hetero-dimer complex formed from a family of five proteins (p50, p52, RelA/p65, RelB, c-Rel) that share a Rel homology domain in their N-terminus and has been implicated in transcriptional regulation during activity-dependent synaptic plasticity (Meberg et al., 1996). Interestingly, while NF-κB has been implicated in the memory consolidation process (Snow et al., 2014), very little is known about how NF-κB mediates transcriptional control of genes that are necessary for proper memory formation and storage in neurons. In the present study, we examined if NF-κB signaling regulates Gadd45 expression and DNA demethylation during the memory consolidation process. Using a combination of pharmacological, genetic, biochemical and molecular approaches, we identified Gadd45β expression and its potential DNA demethylation activity as a novel target for NF-κB activity during hippocampus-dependent memory formation.

## Materials and Methods

### Animals

#### Rats

Male Sprague-Dawley rats (Harlan) weighing 250–300 g at time of arrival were used for these experiments. Animals were single housed in plastic cages, had free access to water and rat chow and were maintained on a 12:12 light:dark cycle. All procedures were approved by the University of Alabama at Birmingham Institutional Animal Care and Use Committee and done in accordance with the National Institute of Health ethical guidelines.

#### c-rel Knockout Mice

The *c-rel*^−/−^ mice were developed as described previously (Ahn et al., 2008). Wild-type (WT) C57BL/6J littermates controls from heterozygote breeding were used as controls.

#### RelA^flox/+^ Mice

A conditionally mutated *p65* (*relA*Δ) mouse line was developed as described previously (Algul et al., 2007). The floxed fragment of *relA* contains exons 7–10, which codes a part of the Rel homology domain and the nuclear localization site. To induce mutation of p65/relA in the CA1 region of the hippocampus, heterozygous mice were anesthetized with an intraperitoneal injection of ketamine-dexmedetomidine and received bilateral injections of Cre-containing (AAV-CMV-Cre-GFP) or empty (AAV-CMV-GFP) viral vectors (Penn Vector Core) 2 weeks prior to behavioral training using stereotaxic coordinates (AP −2.0 mm, ML ± 1.5 mm, DV −1.7 mm) relative to Bregma. The infusion was given over a 10 min period (0.1 μl per minute) for a total volume of 1 μl per side.

### siRNA Delivery

Rats were anesthetized with an intraperitoneal injection of ketamine-dexmedetomidine and received bilateral injections of Accell SMARTpool siRNAs (Thermo) targeting *c-rel* (#E-085667-01-0005) or a negative control (#D-001910-10-05) into the dorsal hippocampus using stereotaxic coordinates (AP −3.6 mm, ML ± 1.7 mm, DV −3.6 mm) relative to bregma. The infusion was given over a 10 min period (0.1 μl per minute) for a total volume of 1 μl per side. Animals were allowed to recover for 5 days before behavioral testing. Fresh Accell siRNA stocks (100 μM) were resuspended in Accell siRNA resuspension buffer to a concentration of ~4.5 μM on the day of surgery.

### Behavioral Procedures

Rats were trained to a standard contextual fear conditioning paradigm in which three shock presentations (0.5 mA, 2 s, 120 s ITI) were given over a 7 min period in a novel context. For latent inhibition, animals were exposed to the training context for 2 h followed immediately by the same 7 min training session described above. In experiments using the NF-κB inhibitor sodium diethyldithiocarbamate trihydrate (DDTC; Sigma), intraperitoneal injections (200 mg/kg in 0.9% Saline) were given 2 h prior to fear conditioning. Mice were trained to a single trial auditory plus contextual fear conditioning paradigm that consisted of a 1.5 min baseline followed a single tone (100 Hz, 30 s)—shock (0.5 mA, 2 s) pairing in a novel context. Testing to the auditory cue occurred the following day and consisted of a 1.5 min baseline followed by a 30 s nonreinforced tone presentation. Two hours after the auditory cue test, animals were placed back into the training context for 3 min to test retention for the contextual cue. Freezing behavior was scored in real-time by Med Associates software.

### Collection of Area CA1

One hour after training, the whole brain was removed and placed in oxygenated (95%/5% O_2_/CO_2_) ice-cold cutting solution (composed of (in mM) 110 sucrose, 60 NaCl, 3 KCl, 1.25 NaH_2_PO_4_, 28 NaHCO_3_, 0.5 CaCl_2_, 7 MgCl_2_, 5 glucose, and 0.6 Ascorbate). The CA1 region of the hippocampus was micro-dissected and flash frozen on dry ice. For the *c-rel* siRNA experiment, brains were rapidly removed and flash frozen on dry ice. The CA1 region of the dorsal hippocampus was then dissected out with the aid of a rat brain matrix (Harvard Apparatus); this was done to collect the area of CA1 targeted by the siRNA infusions. Retrosplenial cortex (RSC) tissue was collected from these animals to confirm diffusion of the siRNA. All isolated tissue was stored at −80°C for future processing.

### Western Blotting

Normalized proteins (3–9 μg) were separated on 7.5% or 20% polyacrylamide gel, transferred onto an Immobilon-FL membrane using a turbo transfer system (Biorad), membranes blocked in Licor blocking buffer and probed with primary antibodies for p65 (1:200, Santa Cruz #SC-372), IκBα (1:200, Santa Cruz #SC-371), c-Rel (1:100, Santa Cruz #SC-71), Actin (1:1000, Abcam #ab1801) and Gadd45β (1:1000, Abcam #ab128920) overnight at 4°C. Secondary goat anti-rabbit 700CW antibody (1:20,000; Licor Biosciences) was used for detection of proteins using the Licor Odyssey system. All protein quantification was done using GeneTools software (Syngene).

### Quantitative RT-PCR

RNA was extracted from isolated CA1 tissue using the All Prep DNA/RNA mini kit (Qiagen), converted to cDNA (iScript cDNA synthesis kit; Biorad) and RT-PCR amplified on the IQ5 or CFX1000 real-time PCR system (Biorad) as described previously (Gupta-Agarwal et al., 2014) with primer annealing temperatures of 59°C for mouse and 62.6°C for rat. Primers were as follows: rat *Gadd45α* (forward: TCATTCGTGCTTTCTGTTGC, reverse: TCCCGGCAAAA ACAAATAAG), rat *Gadd45β* (forward: GAGGGCATGAAGACCAAAAA, reverse: ATTTAGGATGGCCGGGTTAC), rat *Gadd45*γ (forward: GTCCTGAATGTGGACCCTGAC, reverse: ATGGATCTGCAGGGCTATGTC), mouse *Gadd45β* (forward: CTCTTGGGGATCTTCCGTGG, reverse: TGTCGGGGTCCACATTCATC). Quantification of β-*tubulin*-4 levels (rat forward: AGCAACATGAATGACCTGGTG, reverse: GCTTTCCCTAACCTGCTTGG; mouse forward: TAGTGGAGAACACAGACGAGA, reverse: CTGCTGTTCTTACTCTGG ATG) was used as an internal control for normalization. All data was analyzed using the comparative Ct method.

### Chromatin Immunoprecipitation (ChIP)

ChIP was performed as described previously with a small scale modification (Gupta-Agarwal et al., 2014). Briefly, samples were fixed in PBS with 1% formaldehyde, chromatin was sheared using a Bioruptor on high power, lysates centrifuged and diluted in TE and RIPA buffer. Extracts were mixed with MagnaChip magnetic protein A/G beads and immunoprecipitations were carried out at 4°C overnight with primary antibody (anti-Gadd45β) or no antibody (control). Immune complexes were sequentially washed with low salt buffer, high salt buffer, LiCl immune complex buffer and TE buffer, extracted in 1 × TE containing 1% SDS and protein-DNA cross-links were reverted by heating at 65°C overnight. After proteinase K digestion (100 μg; 2 h at 37°C), DNA was extracted by phenol/chloroform/isoamyl alcohol and then ethanol- precipitated. Immunoprecipitated DNA samples were subjected to quantitative real-time PCR using primers specific to the mouse *BDNF* promoter 4 (forward: GCGCGGAATTCTGATTCTGG reverse: AAAGTGGGTGGGAGTCCA). The cumulative fluorescence for each amplicon was taken as a percentage of the input fraction, enrichment over background (no antibody control) calculated and taken as a fold change of the control group.

### Direct Bisulfite Sequencing

Quantification of DNA methylation through direct bisulfite sequencing was performed as described previously (Ryley Parrish et al., 2013). Briefly, 50 μg of genomic DNA was bisulfite treated using the Qiagen Epitech Bisulfite Kit and amplified for a primer targeting 12 CpG sites in the promoter region of rat *BDNF IV* and eight CpG sites in the promoter region of mouse *BDNF IV*. Primer pairs were: rat *BDNF* promoter 4 (forward: GGTAGAGGAGGTATTATATATGATAGTTTA, reverse: TACTCCTATTCTTCAACAAAAAAATTAAAT, product size of 250 base pairs, annealing temperature: 60°C) and mouse *BDNF* promoter 4 (forward: TTATAAAGTATGTAATGTTTTGGAA, reverse: AAATAAAAAAATAAATAAAAATCCAC, product size of 189 base pairs, annealing temperature: 59°C). PCR products were confirmed for size, cleaned using ExoSAP-IT (Affymetrix) and sequenced in duplicate using the reverse primer at the University of Alabama at Birmingham Genomics Core Facility of the Heflin Center for Human Genetics. Using Chromas software to read the electropherogram, the percent methylation of the CpG sites was then determined by the ratio between peak values of guanine (G) and adenine (A) (G/(G+A)).

### Statistical Analyses

All data is presented as group average with the standard error of the mean and was analyzed using Analysis of Variance (ANOVA) with Fisher LSD *post hoc* tests or with student *t*-tests.

## Results

### Isoform-Specific Increases in *Gadd45* Expression in Area CA1 Following Learning

**Figure 1:**
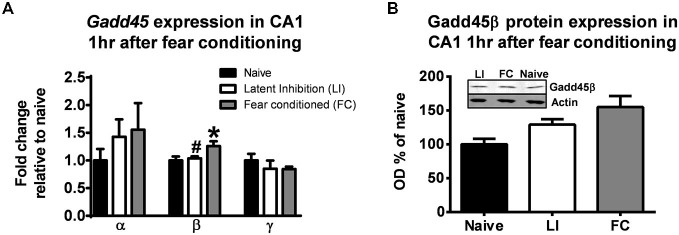
**Gadd45β expression is increased in the hippocampus following learning. (A)** Animals were trained to contextual fear conditioning and area CA1 collected 1 h later (Fear conditioned). A separate group of animals was exposed to the training context for 2 h, followed by the same contextual fear conditioning procedure and area CA1 collected 1 h later (Latent Inhibition). Quantitative RT-PCR revealed an increase in *Gadd45β*, but not *Gadd45α* or *Gadd45*γ, gene expression in area CA1 following fear conditioning (*n* = 5–6 per group). **(B)** Western blot analysis confirmed a moderate increase in Gadd45β protein expression at 1 h in the fear conditioned, but not latent inhibition, group (*n* = 5–6 per group). *p < 0.05 from Naïve. #*p* < 0.05 from FC.

First we tested whether or not learning triggers expression changes of diverse *Gadd45* isoforms. For these experiments, animals were trained in a contextual fear conditioning paradigm and after 1 h area CA1 was isolated and we examined changes in *Gadd45α*, *Gadd45β* and *Gadd45γ* gene expression. We chose to assess *Gadd45* expression levels at 1 h following fear conditioning, as we have previously found optimal changes in DNA methylation levels in area CA1 of the hippocampus (Lubin et al., 2008). As a control for associative memory, we exposed a separate group of animals to a non-associative latent inhibition learning paradigm procedure, which involves exposure to the fear conditioning chamber only (context) followed by a delayed delivery of the aversive footshock 2 h later, preventing the subject to not associate the unconditioned stimulus (footshock) with the conditioned stimulus (context; Gupta et al., 2010). We found significant increases in Gadd45β (*F*_(2,12)_ = 4.067, *p* < 0.05), but not *Gadd45α* (*F*_(2,12)_ = 0.674, *p* = 0.527) or *Gadd45γ* (*F*_(2,11)_ = 0.550, *p* = 0.591) mRNA levels in area CA1 following fear conditioning (Figure 1A). The increase in *Gadd45β* mRNA levels was not present in the latent inhibition group, confirming that *Gadd45β* gene expression changes were specific to context-learning along, and occurred with a moderate increase in Gadd45β protein expression (*F*_(2,10)_ = 3.895, *p* = 0.056; Figure 1B). Collectively, these results suggest that Gadd45β gene and protein expression are increased in area CA1 as a function of associative learning.

### NF-κB Activity is Critical for Increased *Gadd45β* Expression and BDNF Promoter 4 DNA Demethylation Following Learning

**Figure 2:**
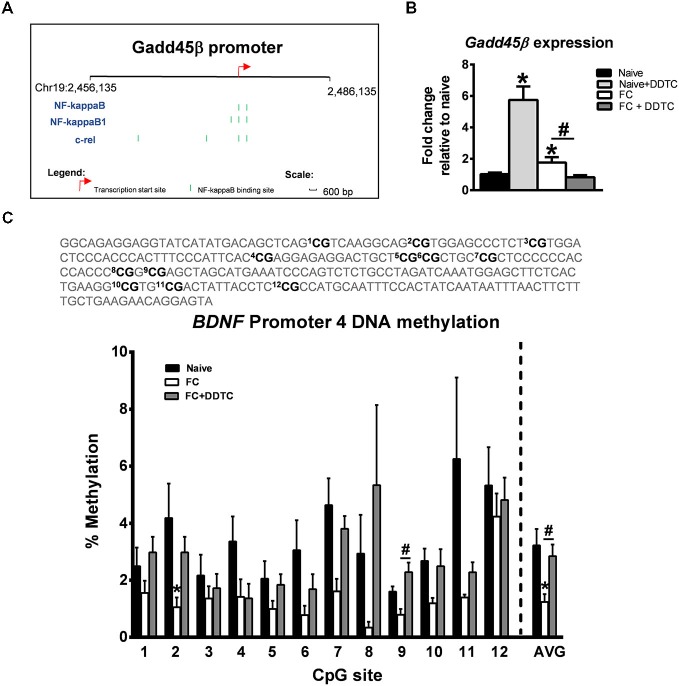
**Inhibition of NF-κB activity abolishes learning-induced changes in *Gadd45β* expression and *BDNF* DNA demethylation in the hippocampus. (A)** *In silico* analysis revealed several NF-κB consensus sequences in the *Gadd45β* promoter. **(B)** Pharmacological inhibition of NF-κB activity with DDTC abolished fear conditioning-induced increases in *Gadd45β* expression in area CA1 (*n* = 3–6 per group). **(C)** Bisulfite sequencing analysis of CpG sites in the rat *BDNF* Promoter 4 region revealed that inhibition of NF-κB with DDTC completely prevented the fear conditioning-induced decreases in *BDNF* Promoter 4 DNA methylation in area CA1 (*n* = 4–6 per group). *p < 0.05 from Naïve. ^#^*p* < 0.05 from FC.

Considering that NF-κB is a transcription factor that plays a critical role in memory formation (Yeh et al., 2002; Lubin and Sweatt, 2007; Federman et al., 2013), we next determined whether or not *Gadd45β* expression was being regulated by NF-κB transcriptional activity during the memory consolidation period. *In silico* analysis revealed several NF-κB consensus sequences within the *Gadd45β* gene (Figure 2A). We found that pharmacological inhibition of NF-κB signaling activity with diethyldithiocarbamate (DDTC) abolished the fear conditioning-induced increases in *Gadd45β* expression in area CA1 (*F*_(3,11)_ = 34.47, *p* < 0.001; Figure 2B), suggesting that NF-κB signaling was critical for the increased transcription of *Gadd45β* following learning. Since *Gadd45β* regulates DNA demethylation during memory formation, we next tested if pharmacological blockade of NF-κB activity with DDTC prevented DNA demethylation of the *BDNF* gene, a well-established regulator of memory formation that undergoes dynamic activity-dependent and promoter-specific changes in DNA methylation levels (Lee et al., 2004; Bekinschtein et al., 2007; Lubin et al., 2008; Peters et al., 2010). Remarkably, we found that while fear conditioning resulted in decreased *BDNF* Promoter 4 DNA methylation, inhibiting NF-κB activity completely attenuated this effect (*F*_(2,12)_ = 4.589, *p* < 0.05; Figure 2C). Together, these results suggest that NF-κB controls *Gadd45β* expression and* BDNF* DNA demethylation in the hippocampus during memory formation.

### Conditional Mutation of *p65/RelA* Impairs Memory Formation but does not Alter *Gadd45β* Expression

**Figure 3:**
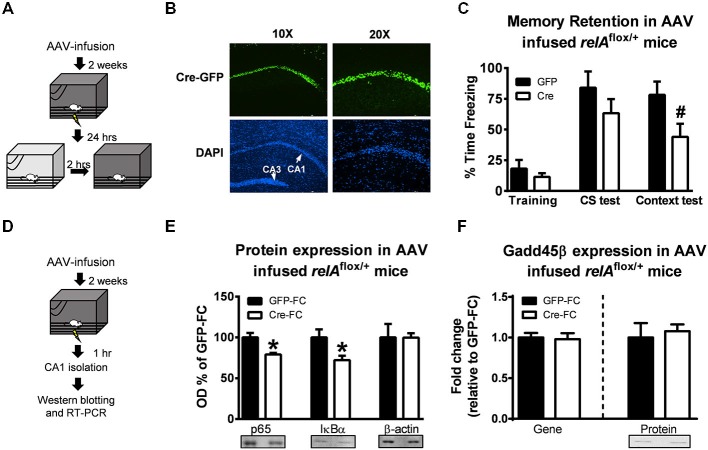
**Conditional mutation of *p65/relA* does not alter *Gadd45*β expression in the hippocampus following learning. (A)** Floxed *p65/relA* mice (*relA*^flox/+^) were injected with Cre-containing (AAV-CMV-Cre-GFP) or empty (AAV-CMV-GFP) adenoviruses 2 weeks prior to fear conditioning to induce mutation in the *p65/relA* gene. The day after training, mice were tested to the auditory cue followed by the context 2 h later. **(B)** Immunohistochemistry showing GFP expression in the hippocampus. DAPI was used to visualization the different hippocampus sub-regions. **(C)** While there were no differences between group during training or testing to the auditory cue, *relA*^flox/+^ mice receiving Cre injections had impaired contextual fear memory relative to controls (*n* = 5–7 per group). **(D)** *relA*^flox/+^ were injected with Cre-containing (AAV-CMV-Cre-GFP) or empty (AAV-CMV-GFP) adenoviruses 2 weeks prior to fear conditioning and area CA1 collected 1 h later. **(E)** Western blot analysis confirmed knockdown of p65 and IκBα expression in the Cre-infused mice (*n* = 4–5 per group). **(F)** There were no differences in Gadd45β gene or protein expression following Cre-infusion relative to GFP-infused controls (*n* = 4–5 per group). *p < 0.05 from GFP. ^#^*p* = 0.054 from GFP.

While our pharmacological manipulation with DDTC suggests a role for NF-κB activity in regulation *Gadd45β* expression and *BDNF* DNA demethylation in the hippocampus during memory formation, our studies do not yet distinguish between the contributions of different NF-κB subunits that may have been involved. The p65/RelA and p50 heterodimer is critical for nuclear translocation and activation of the NF-κB complex, thus we tested if manipulation of p65/RelA would mimic the effects of inhibiting NF-κB signaling activity with DDTC on *Gadd45β* expression following learning. We conditionally mutated *p65/relA* using a Cre-loxP insert spanning exons 7–10 containing the Rel homology domain and nuclear translocation site (Algul et al., 2007). To induce the *relA*Δ mutation in area CA1 of the adult hippocampus, we infused Cre-containing (AAV-CMV-Cre-GFP) or empty (AAV-CMV-GFP) viral vectors 2 weeks prior to fear conditioning (Figures 3A,B). Consistent with a role for NF-κB signaling in hippocampus-dependent memory formation, we found that while *relA*^flox/+^ mice successfully acquired the fear memory during training (*t*_(10)_ = 0.9807, *p* = 0.349), when tested 24 h later, *relA*^flox/+^ mice had significant impairments in memory retention for contextual (*t*_(10)_ = 2.182, *p* = 0.054) but not hippocampus-independent auditory (*t*_(9)_ = 1.184, *p* = 0.266), fear memory (Figure 3C). This is the first evidence that local knockdown of *p65/relA* in the adult hippocampus impairs long-term memory formation. Next, we tested if mutation of *p65/relA* altered *Gadd45β* expression following learning (Figure 3D). Interestingly, while we confirmed that *relA*^flox/+^ mice had reduced expression of p65 (*t*_(6)_ = 3.583, *p* < 0.05) and the NF-κB associated protein IκBα (*t*_(7)_ = 2.624, *p* < 0.05) relative to controls (Figure 3E), we found no effect of the *p65/relA* mutation on *Gadd45β* mRNA (*t*_(6)_ = 0.214, *p* = 0.837) or protein (*t*_(7)_ = 0.427, *p* = 0.681) levels following learning (Figure 3F). Thus far, our results suggest that while NF-κB signaling and p65/RelA activity in the hippocampus are critical for memory formation, p65/RelA is not responsible for the NF-κB-dependent regulation of *Gadd45β* expression during the memory consolidation process.

### Manipulation of c-Rel in Area CA1 Prevents Increases in *Gadd45β* Expression and *BDNF* Promoter 4 DNA Demethylation Following Learning

**Figure 4:**
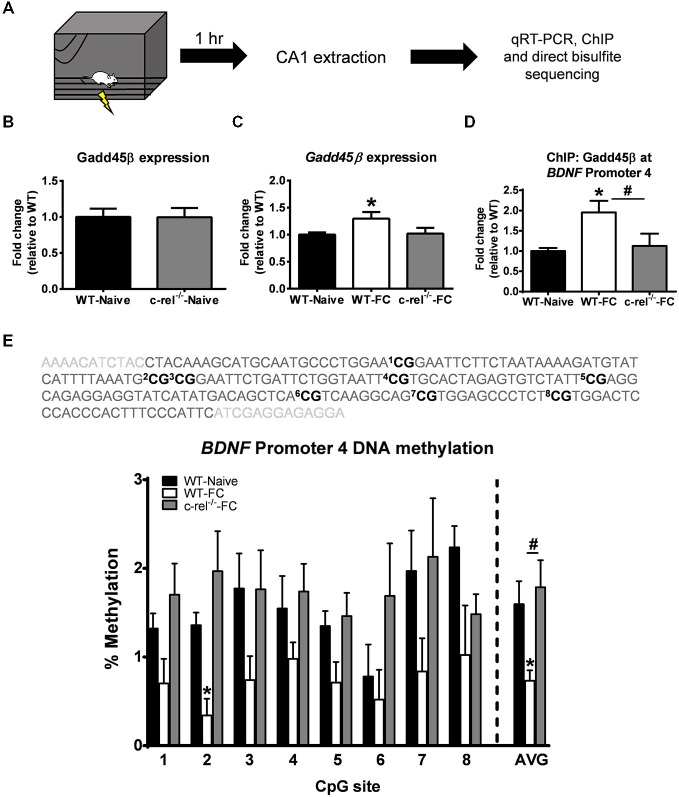
**Knockout of *c-rel* prevents fear conditioning-induced changes in *Gadd45β* expression and *BDNF* DNA methylation in the hippocampus. (A)** Wild-type (WT) or *c-rel* knockout (*c-rel^−/−^*) mice were fear conditioned and area CA1 collected 1 h later. **(B)** Basal expression of *Gadd45β* was not altered in *c-rel*^−/−^ mice (*n* = 4–5 per group). **(C)** *Gadd45β* expression was increased in WT, but not *c-rel*^−/−^, mice following fear conditioning (*n* = 8 per group). **(D)** Chromatin immunoprecipitation revealed an increase in Gadd45β binding at *BDNF* Promoter 4 following fear conditioning, which was lost in *c-rel*^−/−^ mice (*n* = 4 per group). **(E)** Bisulfite sequencing analysis of CpG sites in the mouse *BDNF* Promoter 4 region revealed a fear conditioning-induced decrease in *BDNF* Promoter 4 DNA methylation in area CA1 that was prevented in *c-rel*^−/−^ mice (*n* = 3–4 per group). *p < 0.05 from WT. ^#^*p* < 0.05 from WT-FC.

In our *in silicio* analysis we found multiple c-Rel consensus sites in the *Gadd45β* promoter, suggesting that c-Rel containing NF-κB complexes may be responsible for the learning-dependent increases in *Gadd45β* expression in area CA1. To test this, we examined *Gadd45β* expression in *c-rel* knockout (*c-rel*^−/−^) mice (Figure 4A), which we have previously shown to have impaired hippocampus-dependent but not hippocampus-independent fear memory (Levenson et al., 2004; O’Riordan et al., 2006; Ahn et al., 2008). First, we examined if a loss of *c-rel* during development resulted in long-term changes in *Gadd45β* expression. However, we did not observe altered basal levels of *Gadd45β* expression in area CA1 (*t*_(7)_ = 0.022, *p* = 0.982; Figure 4B) of *c-rel*^−/−^ mice, suggesting normal *Gadd45β* expression in the hippocampus. Next, we tested if *c-rel*^−/−^ mice have altered *Gadd45β* expression in the hippocampus in response to learning. We found an increase in *Gadd45β* expression in fear conditioned WT mice relative to naïve controls (*t*_(14)_ = 2.256, *p* < 0.05). Surprisingly, this increase in *Gadd45β* mRNA levels were not present in *c-rel*^−/−^ mice (*t*_(14)_ = 0.177, *p* = 0.862), suggesting that c-Rel containing NF-κB complexes were critical for the NF-κB-dependent regulation of *Gadd45β* expression following learning (Figure 4C). Since Gadd45β regulates DNA demethylation and we found that pharmacological inhibition of NF-κB signaling activity with DDTC prevented learning-induced DNA demethylation of *BDNF* Promoter 4, we next tested if *BDNF* Promoter 4 DNA demethylation was altered in *c-rel*^−/−^ mice. Using chromatin immunoprecipitation, we found an increase in Gadd45β protein levels at *BDNF* Promoter 4 in area CA1 following fear conditioning that was abolished in *c-rel*^−/−^ mice (*F*_(2,9)_ = 4.543, *p* < 0.05; Figure 4D) associated with *BDNF* Promoter 4 DNA demethylation (*F*_(2,7)_ = 4.504, *p* = 0.055), suggesting that there is a loss of learning-dependent Gadd45β accumulation at the *BDNF* gene in the hippocampus of *c-rel* knockout mice. Remarkably, we found that while fear conditioning resulted in decreased *BDNF* Promoter 4 DNA methylation relative to controls (*t*_(4)_ = 3.039, *p* < 0.05), this did not occur in *c-rel*^−/−^ mice (*t*_(5)_ = 0.454, *p* = 0.668; Figure 4E). This suggests that c-Rel is likely responsible for the NF-κB-dependent regulation of *Gadd45β* expression and *BDNF* Promoter 4 DNA demethylation during memory formation.

**Figure 5:**
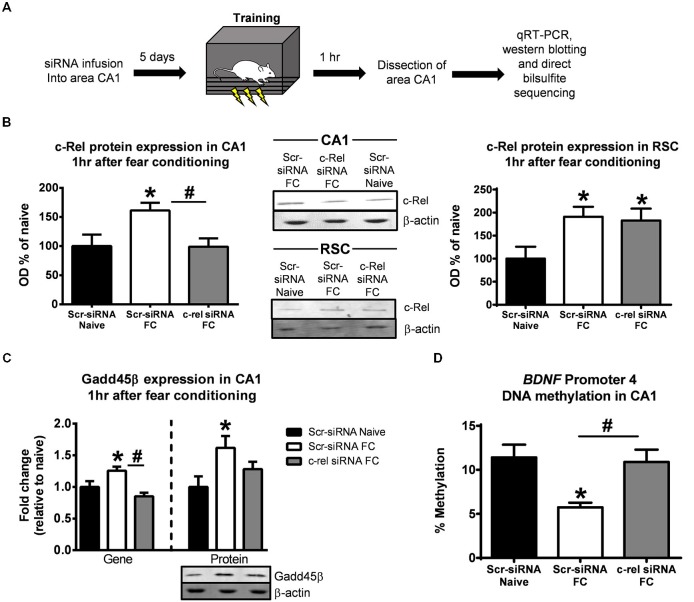
**Local knockdown of *c-rel* in the hippocampus prevents fear conditioning-induced changes in *Gadd45β* expression and *BDNF* DNA methylation. (A)** Rats were infused Accell siRNAs against *c-rel* or a negative control (Scr-siRNA) 5 days prior to fear conditioning and area CA1 collected 1 h later. **(B)** Fear conditioning increased c-Rel protein expression in the hippocampus which was blocked by the *c-rel* siRNA, confirming effective gene knockdown by the siRNA infusion (left). Fear conditioning-induced increases in c-Rel protein expression in the retrosplenial cortex (RSC) were not altered by the *c-rel* siRNA when infused in the hippocampus, confirming that the siRNA did not diffuse up the injection tract (right). **(C)** Knockdown of *c-rel* prevented the fear conditioning induced increases in Gadd45β gene (*n* = 3–4 per group) and protein expression (*n* = 5 per group) in the hippocampus. **(D)** Knockdown of *c-rel* prevented the fear conditioning induced decreases in rat *BDNF* Promoter 4 DNA methylation in the hippocampus (*n* = 3 per group). *p < 0.05 Scr-siRNA Naïve. ^#^*p* < 0.05 from Scr-siRNA FC.

An alternative explanation for the effects described above is that the alterations in *Gadd45β* expression and *BDNF* DNA demethylation in area CA1 of *c-rel*^−/−^ mice are due to the loss of *c-rel* in multiple brain regions simultaneously, which could result in wide-scale epigenetic changes across the neural circuit. To test this possibility, we locally knocked-down *c-rel* in area CA1 of the hippocampus in adult animals using siRNA technology and examined changes in *Gadd45β* expression and *BDNF* Promoter 4 DNA demethylation following learning (Figure 5A). First, we confirmed the effectiveness of our siRNA by examining c-Rel protein expression in area CA1 and the surrounding cortical region following fear conditioning (Figure 5B). In area CA1, we found that fear conditioning increased c-Rel expression, which was attenuated in *c-rel* siRNA infused animals (*F*_(2,10)_ = 4.150, *p* < 0.05). However, in the RSC, which requires *de novo* protein synthesis for the consolidation of contextual fear memories (Kwapis et al., 2015), we found a learning-induced increase in c-Rel expression that was unaffected by infusion of the *c-rel* siRNA into the hippocampus (*F*_(2,10)_ = 3.838, *p* = 0.058). These results suggest that our siRNA effectively targeted *c-rel* in the hippocampus and supports previous studies that found increased expression of NF-κB proteins during enhanced synaptic activity (Meberg et al., 1996). Next, we examined what effect *c-rel* knockdown had on Gadd45β expression and DNA demethylation following fear conditioning. We found that siRNA-mediated knockdown of *c-rel* in adulthood completely abolished the learning-induced increases in *Gadd45β* gene (*F*_(2,7)_ = 8.760, *p* < 0.05) and largely reduced the increases in protein expression (*F*_(2,12)_ = 3.762, *p* = 0.053) in area CA1 (Figure 5C), confirming what we observed in *c-rel*^−/−^ mice. Additionally, *c-rel* siRNA knockdown abolished the learning-induced decreases in *BDNF* Promoter 4 DNA methylation (*F*_(2,6)_ = 6.786, *p* < 0.05; Figure 5D). In combination with our *c-rel*^−/−^ mice data, these results strongly suggest that c-Rel regulates *Gadd45β* expression and *BDNF* Promoter 4 DNA demethylation in the hippocampus during memory consolidation.

## Discussion

Several studies have implicated DNA demethylation mechanisms in the memory consolidation process (Kaas et al., 2013; Rudenko et al., 2013; Li et al., 2014), however, the mechanisms regulating this process have remained equivocal. In the present study, we found that learning in an associative fear conditioning paradigm dynamically regulates the expression of *Gadd45β* in the hippocampus, which is known to regulate DNA demethylation changes that are critical for various forms of synaptic plasticity. Importantly, for the first time we identified the NF-κB transcription pathways as a key regulator of *Gadd45β* expression and DNA demethylation during the memory consolidation process. Remarkably, the loss of *Gadd45β* expression and DNA demethylation in the hippocampus observed following pharmacological inhibition of NF-κB prior to fear conditioning could be completely mimicked by knockout or knockdown of *c-rel*, but not *relA/p65*, expression. Collectively, these findings suggest that c-Rel may be a critical regulator of Gadd45β-mediated DNA demethylation during the memory consolidation process and strongly support a novel epigenetic role for NF-κB signaling in DNA demethylation mechanisms during memory formation in the hippocampus.

The NF-κB transcription factor has been widely implicated in synaptic plasticity and memory formation in neurons (Snow et al., 2014). In terms of its role in memory formation, numerous studies have reported memory impairments following global or region-specific inhibition of NF-κB activity in a diverse group of organisms and transcription of several genes has been shown to be dependent on NF-κB activation, such as *zif268/egr1* and *Arc* (O’Mahony et al., 2006; Zalcman et al., 2015), which have well described roles in the memory consolidation process (Guzowski et al., 2001; Hall et al., 2001; Ploski et al., 2008). Additionally, several studies have identified epigenetic functions of NF-κB activity in memory formation, particularly in the regulation of histone acetylation processes (Lubin and Sweatt, 2007; Si et al., 2012; Federman et al., 2013). In the present study, we add to this growing number of transcriptional processes regulated by NF-κB by demonstrating a role for it in activity-dependent DNA demethylation. However, unlike the other transcriptional processes described above which are generally transient, this DNA methylation function of NF-κB has the potential to be a long-term mechanism for gene regulation since DNA methylation can persistent across the lifespan and between generations (Roth et al., 2009; Dias and Ressler, 2014). Thus, NF-κB-dependent regulation of *Gadd45β* expression and DNA demethylation may represent a mechanism for both memory formation and maintenance in the hippocampus.

While it has been known for several years that NF-κB activity is critical for synaptic plasticity and memory formation, few studies have examined the specific contribution of individual NF-κB subunits to transcriptional regulation during the memory consolidation process. The most studied subunit has been p50 since the p65/relA and p50 heterodimer is critical for nuclear translocation and activation of the NF-κB complex. In general, a loss of the p50 subunit impairs hippocampus-dependent synaptic plasticity and memory formation, though the results have been mixed (Kassed et al., 2002; Kassed and Herkenham, 2004; Denis-Donini et al., 2008; Lehmann et al., 2010; Oikawa et al., 2012). Additionally, it has previously been shown that a loss of the c-rel subunit impairs hippocampal LTP and memory formation (Levenson et al., 2004; O’Riordan et al., 2006; Ahn et al., 2008), suggesting that the c-Rel subunit is a critical regulator of learning-dependent synaptic plasticity (O’Riordan et al., 2006). However, no study, to date, has directly compared the contribution of individual NF-κB subunits to the regulation of a specific transcriptional process critical for synaptic plasticity and memory formation. Thus, our result that c-Rel, but not RelA/p65, regulates Gadd45 expression and DNA demethylation is the first evidence to implicate unique transcriptional functions of the NF-κB subunits during the memory consolidation process. Future studies should aim to examine the contribution of the different NF-κB subunits to other learning-dependent transcriptional processes.

DNA demethylation mechanisms have been implicated in synaptic plasticity and memory formation (Kaas et al., 2013; Rudenko et al., 2013; Li et al., 2014; Feng et al., 2015), however, little is known about how this process is regulated following learning. *Gadd45β* is one mechanism controlling activity-dependent DNA demethylation in neurons (Barreto et al., 2007; Ma et al., 2009; Niehrs and Schäfer, 2012), though how *Gadd45β* expression is regulated during memory formation remains unknown. In the present study, we identified NF-κB as the first regulator of *Gadd45β* transcription in the hippocampus during the memory consolidation process, though we do not know if c-Rel directly regulates Gadd45 expression or if it does so indirectly through other signaling pathways. However, a loss of NF-κB signaling also prevented learning-dependent DNA demethylation, revealing a novel epigenetic role for NF-κB signaling in memory formation. Interestingly, this is the second identified epigenetic function of NF-κB during memory formation, as previous studies from our group and others have suggested that NF-κB regulates histone acetylation during the memory storage process (Lubin and Sweatt, 2007; Si et al., 2012; Federman et al., 2013). Considering that very little is currently known about how different epigenetic mechanisms are regulated during memory formation and storage (Jarome and Lubin, 2014), these studies collectively suggest that NF-κB might be a critical regulator of different epigenetic marks during memory consolidation, however, at this point its role is exclusively in gene activation rather than repression. Future studies should focus on identification of other potential epigenetic functions of NF-κB during the memory consolidation process.

In summary, we found that NF-κB activity is a critical regulator of *Gadd45β* expression and active *BDNF* DNA demethylation in the hippocampus during the memory consolidation process. Importantly, the NF-κB-dependent regulation of *Gadd45β* and DNA demethylation were controlled by the c-Rel, but not RelA/p65, subunit of the NF-κB complex, suggesting that c-Rel was critical for learning-dependent DNA demethylation at least at the *BDNF* gene in the hippocampus. These findings identify a novel role for NF-κB/c-Rel in activity-dependent synaptic plasticity and suggest that DNA demethylation may be largely controlled through NF-κB-dependent signaling during the memory consolidation process.

## Conflict of Interest Statement

The authors declare that the research was conducted in the absence of any commercial or financial relationships that could be construed as a potential conflict of interest.
